# Conceptual Design of a Universal Donor Screening Approach for Vaginal Microbiota Transplant

**DOI:** 10.3389/fcimb.2019.00306

**Published:** 2019-08-28

**Authors:** Kevin DeLong, Sabrine Bensouda, Fareeha Zulfiqar, Hannah C. Zierden, Thuy M. Hoang, Alison G. Abraham, Jenell S. Coleman, Richard A. Cone, Patti E. Gravitt, Craig W. Hendrix, Edward J. Fuchs, Charlotte A. Gaydos, Ethel D. Weld, Laura M. Ensign

**Affiliations:** ^1^The Center for Nanomedicine, The Wilmer Eye Institute, Johns Hopkins University School of Medicine, Baltimore, MD, United States; ^2^Department of Ophthalmology, The Wilmer Eye Institute, Johns Hopkins University School of Medicine, Baltimore, MD, United States; ^3^Division of Clinical Pharmacology, Department of Medicine, Johns Hopkins University School of Medicine, Baltimore, MD, United States; ^4^Department of Chemical and Biomolecular Engineering, Johns Hopkins University, Baltimore, MD, United States; ^5^Department of Pharmacology and Molecular Sciences, Johns Hopkins University School of Medicine, Baltimore, MD, United States; ^6^Department of Epidemiology, Johns Hopkins Medical Institutions, Baltimore, MD, United States; ^7^Department of Gynecology and Obstetrics, Johns Hopkins University School of Medicine, Baltimore, MD, United States; ^8^Department of Biophysics, Johns Hopkins University, Baltimore, MD, United States; ^9^Department of Global Health, George Washington University, Washington, DC, United States; ^10^Division of Infectious Diseases, Department of Medicine, Johns Hopkins University School of Medicine, Baltimore, MD, United States

**Keywords:** microbiota, fecal microbiota transplant (FMT), bacterial vaginosis (BV), *Lactobacillus*, urinary tract infection (UTI), cervicovaginal secretions (CVS), sexually transmitted infections

## Abstract

The success of fecal microbiota transplant (FMT) in treating recurrent *Clostridioides difficile* infection has led to growing excitement about the potential of using transplanted human material as a therapy for a wide range of diseases and conditions related to microbial dysbiosis. We anticipate that the next frontier of microbiota transplantation will be vaginal microbiota transplant (VMT). The composition of the vaginal microbiota has broad impact on sexual and reproductive health. The vaginal microbiota in the “optimal” state are one of the simplest communities, dominated by one of only a few species of Lactobacillus. Diversity in the microbiota and the concomitant depletion of lactobacilli, a condition referred to as bacterial vaginosis (BV), is associated with a wide range of deleterious effects, including increased risk of acquiring sexually transmitted infections and increased likelihood of having a preterm birth. However, we have very few treatment options available, and none of them curative or restorative, for “resetting” the vaginal microbiota to a more protective state. In order to test the hypothesis that VMT may be a more effective treatment option, we must first determine how to screen donors to find those with minimal risk of pathogen transmission and “optimal” vaginal microbiota for transplant. Here, we describe a universal donor screening approach that was implemented in a small pilot study of 20 women. We further characterized key physicochemical properties of donor cervicovaginal secretions (CVS) and the corresponding composition of the vaginal microbiota to delineate criteria for inclusion/exclusion. We anticipate that the framework described here will help accelerate clinical studies of VMT.

## Introduction

Evidence continues to accumulate that demonstrates the critical role that bacteria play in human health and disease. It is often cited that the commensal bacteria colonizing our epithelial surfaces, glands, and fluids outnumber the cells that make up our bodies (Abbott, 2016; Sender et al., 2016). It has even been suggested that the gut microbiota should be considered an additional organ (Baquero and Nombela, 2012; Liu, 2016). The gut microbiota is perhaps the most extensively characterized, and has been shown to influence a wide range of diseases and disorders affecting the gut and beyond (Clemente et al., 2012; Valdes et al., 2018). Indeed, the premise of microbiota transplantation originated in the gut with the development of fecal microbiota transplantation (FMT) as a strategy for treating recurrent *Clostridioides difficile* (*C. difficile*) infection. The tremendous clinical success of FMT has led to explorations in using FMT for treating inflammatory bowel disease, obesity, liver disease, depression, food allergies, antibiotic resistance, malnutrition, multiple sclerosis, and more (Borody et al., 2013). Further, FMT has motivated the study of other forms of microbiota transfer, including skin microbiota transplant (Myles et al., 2018; Perin et al., 2018) and vaginal microbiota transfer from mother to babies born by Cesarean section (Dominguez-Bello et al., 2016).

Despite being one of the simpler commensal bacteria communities, cervicovaginal microbiota play a key role in sexual and reproductive tract health. The earliest attempts to characterize the cervicovaginal microbiota were conducted in the mid 1800s, a time when puerperal sepsis killed at least 15% of women giving birth in Europe and America (Hallett, 2005). In 1879, Louis Pasteur reported observation of streptococci in the blood of women with puerperal fever (Dunn, 2005). He believed this may have resulted from mechanical trauma that allowed bacteria in the vagina to enter the bloodstream. Later in 1892, Doderlein reported that women who had Gram-positive, lactic acid-producing bacilli in their vagina at the time of childbirth were less likely to develop puerperal sepsis after delivery (Doderlein, 1892; Thomas, 1928). Although there has been increasing awareness of the broad spectrum of “normal” (Smith and Ravel, 2017; Anahtar et al., 2018), it is generally considered that the “optimal” vaginal microbiota communities are dominated by one of only a handful of species of *Lactobacillus* (Linhares et al., 2011; Petrova et al., 2015). Diversity in the vaginal microbiota and lack of dominance by *Lactobacillus* species can be described clinically as bacterial vaginosis (BV), a condition that has been linked to increased risk of sexually transmitted infection acquisition and transmission (Cherpes et al., 2003; Wiesenfeld et al., 2003; Allsworth et al., 2008; Atashili et al., 2008; Cohen et al., 2012), urinary tract infections (Sumati and Saritha, 2009; Stapleton, 2016), and fertility and pregnancy outcomes (Hyman et al., 2014; Petricevic et al., 2014; García-Velasco et al., 2017; Stout et al., 2017). Further, rates of BV relapse after standard antibiotic treatment can be as high as 70% within 3 months (Larsson and Forsum, 2005). Beyond BV, vaginal microbiota have also been implicated in recurrent yeast infections (Zhou et al., 2009; Liu et al., 2013), colonization with group B streptococcus (Rosen et al., 2017; van de Wijgert, 2017), and potentially reproductive tract cancers (Xu et al., 2014; Kyrgiou et al., 2017). Many have drawn attention to the limitations of currently available treatments for BV and the need for innovation in approaches for modifying the vaginal microbiota (Bradshaw and Sobel, 2016; Martin and Marrazzo, 2016).

Vaginal microbiota transplant (VMT) has the potential to revolutionize the way we view and treat conditions affecting the female reproductive tract. Unfortunately, study of vaginal microbiota in preclinical animal models is severely limited by the fact that dominance of the vaginal microbiota and acidification by *Lactobacillus* species is a uniquely human phenomenon (Miller et al., 2016; Witkin and Linhares, 2017). Even our primate cousins have low vaginal colonization by *Lactobacillus* species, and it has been suggested that the normal rhesus macaque vaginal microbiota is a good model for human BV (Spear et al., 2010; Mirmonsef et al., 2012; Yildirim et al., 2014). Unlike FMT, there is no previous history of anecdotal clinical implementation of VMT. However, there is significant epidemiological evidence of vaginal microbiota transfer between women who have sex with women (WSW) (Marrazzo et al., 2002, 2009; Vodstrcil et al., 2015). Thus, a logical next step is to determine whether cervicovaginal secretions (CVS) can be used to transplant vaginal microbiota from a donor to a recipient in a clinical setting. Undoubtedly, ensuring safety and tolerability are top priorities. Here, we describe the development of a universal donor screening protocol intended for maximal risk reduction to mitigate potential transmission of infectious pathogens, as well as collection, characterization, and testing procedures for donor CVS.

## Materials and Methods

### Ethics Statement

The sample collection and testing procedures described here were approved by the Johns Hopkins University Institutional Review Boards as a part of study IRB00131437. Informed consent was obtained from all human subjects prior to participation.

### Screening Questionnaire

We have developed a questionnaire for pre-screening potential VMT donors. The questionnaire includes all questions listed in the FDA Guidance for Industry for Eligibility Determination for Donors of Human Cells, Tissues, and Cellular and Tissue-Based Products (HCT/Ps) (Section IV, Donor screening, §1271.75, section E). In addition, we included screening questions that are consistent with what has been observed to impact vaginal microbiota and stability of vaginal microbiota communities, including sexual history, sexual behavior, and vaginal product usage. We also included questions about medical history, and travel history (e.g., potential exposure to Zika or Ebola) that could have an impact on risk of incident sexually transmitted infections. For the pilot donor screening study described herein, we used an abbreviated questionnaire with the primary goal of correlating testing outcomes with self-reported sexual behavior, vaginal symptoms, vaginal product usage, and self-reported history of reproductive tract and sexually transmitted infections. Participant demographics and questionnaire data can be found in Table 1.

**Table 1:** Participant demographics and questionnaire data^*^.

Age	Median (range)
	26.5 (23–35)
Ethnicity	Number (%)
Hispanic or Latino Not Hispanic or Latino	2 (10) 18 (90)
Race	Number (%)
White Asian White/Asian Native Hawaiian or other Pacific Islander Other	12 (60) 3 (15) 2 (10) 1 (5) 2 (10)
Type of Birth Control	Number (%)
None Condoms Oral Contraceptive IUD Copper IUD Progestin IUD	4 (20) 2 (10) 5 (25) 9 (45) 3 (15) 6 (30)
Reported Symptoms	Number (%)
None Staining of underwear Vaginal Odor Vaginal Discharge Vaginal Itch	15 (75) 2 (10) 1 (5) 2 (10) 1 (5)
Previous Conditions	Number (%)
Yeast Infection HPV Bacterial Vaginosis Vaginal Irritation Vaginal Itch (Persistent) UTI Abdominal or Pelvic Pain Chlamydia Herpes	12 (60) 1 (5) 2 (10) 1 (5) 1 (5) 1 (5) 1 (5) 3 (15) 1 (5)
Products Used	Number (%)
None Vaginal Douche Feminine towelettes Boric Acid	16 (80) 1 (5) 2 (10) 1 (5)
Number of sexual partners (lifetime)	Median (range)
Men Women	6.5 (0–29) 0 (0–2)
Number of sexual partners in the last month	Median (range)
Men Women	0 (0–1) 0 (0)
Is the current male partner circumcised?	Number (%)
Yes No No current male partner	15 (75) 3 (15) 2 (10)
Tobacco use	Number (%)
No	20 (100)
Have you ever given birth to a baby	Number (%)
No	20 (100)

* *The following symptoms “you currently have” were not selected by any participant: pain during intercourse; abdominal or pelvic pain; vaginal irritation; pain during urination. The following options for conditions that “you have ever been diagnosed with” were not selected by any participant: trichomoniasis, gonorrhea, syphilis, pelvic inflammatory disease, other please specify. The following options for product use within the past 6 months were not selected by any participant: feminine hygiene spray, feminine hygiene powder, norforms, vaginal acid gel*.

### Test List

The list of clinical tests and laboratory characterizations with their submeasures and readouts with normal ranges, if applicable, can be found in Supplementary Tables 1 and 2, respectively. We followed the FDA Guidance for Industry for Eligibility Determination for Donors of Human Cells, Tissues, and Cellular and Tissue-Based Products (HCT/Ps) (Section VI, Donor Testing, §1271.85, sections A and B) for testing of leukocyte-rich cells or tissues, which also required the use of a FDA-licensed, cleared, or approved donor screening test where such a test is available (Memorial Blood Centers, St. Paul, MN). Additional testing was conducted by Johns Hopkins Medical Institutions Medical Laboratories, including tests for herpes viruses, hepatitis A, *Toxoplasma gondii*, Epstein-barr virus (EBV), rubella virus, general immunocompetence, pregnancy, and bacterial and fungal cultures. Several tests were subsequently sent to Quest Diagnostics, as indicated by test codes beginning in “Q” in Supplementary Table 1. It was noted after the first four participants that many did not recall their hepatitis A vaccination status while testing positive for hepatitis A IgG, so a test for hepatitis A IgM was added. Additional laboratory characterizations for sexually transmitted infections, included nucleic-acid amplification tests (NAAT) for *Chlamydia trachomatis, Neisseria gonorrhoeae, Trichomonas vaginalis*, and *Mycoplasma genitalium*, and Human papilloma viruses (HPV), as well as quantitative polymerase chain reaction (qPCR) and 16S rDNA sequencing to determine composition of vaginal microbiota communities. As 16S rDNA sequencing runs typically require batching of samples into a 96 well plate format, we sought to develop a qPCR approach that was predictive of the relative abundance of *Lactobacillus* species and *Gardnerella vaginalis* and could be readily performed on individual samples on the same day for rapid screening and eligibility determination.

### Sample Collection

Potential study participants were recruited from a list of participants from the investigator's prior studies that (i) had agreed to be contacted for potential participation in future studies, and (ii) had provided *Lactobacillus*-dominated CVS samples as part of these prior studies. To be included in the study, all participants identified as female and were pre-menopausal between the ages of 18–45 yrs. The potential participants were informed that at the time of sample collection, they must not be currently menstruating or within 3 days of their last menstrual period, they must be currently healthy and free of vaginal symptoms, and must not have used vaginal products or had vaginal intercourse in the prior 3 days. Participants were advised to drink extra water in the time leading up to their appointment to ensure proper hydration for blood collection. Participants were consented using an interactive question and answer approach that explained the purpose of the study, the associated risks, and the testing information that was to be obtained. The participants signed a consent form for HIV testing in the state of Maryland. The participants then filled out the abbreviated questionnaire and asked questions for clarification as needed. The participants were then given a bag containing 5 unwrapped vaginal swabs [2 × BD Eswabs (Becton Dickinson), 1 × Mini-tip flocked swabs with viral transport media (Becton Dickinson), 1 × Digene HC2 DNA collection brushes (Qiagen), 1 × Aptima vaginal swab (Hologic)], a Softdisc menstrual fluid collection device (The Flex Company), a sterile wrapped urine specimen cup, and a 50 mL conical tube (Corning Falcon). The participants were shown diagrams to instruct them on how to use each swab and how to insert the Softdisc to collect CVS using a previously described method (Boskey et al., 2003). It was emphasized that the participants take their time collecting each specimen carefully, to ensure that none of the materials touched unintended surfaces, and to return for replacement swabs if any were dropped or contacted a surface other than the vaginal wall. The participant then went to a self-locked single stall restroom for sample self-collection. Upon their return to the clinical area, the participant was prepared for blood collection. The total volume of blood collected across various tubes to perform all tests was ~50 mL. Specimens were then grouped for immediate overnight shipping to Memorial Blood Centers, transport to the JHMI Medical Laboratories, and transport on ice to the respective laboratory facilities for testing and characterization. Three participants had menstrual blood in their CVS, either trace amounts still present despite reporting 3 days post the end of their menstrual period, or because of an unanticipated early start to their period. They returned within 2–20 days to provide another CVS sample and vaginal Eswab for 16S rDNA sequencing, which were the samples used for the data reported herein.

### Laboratory Characterizations

#### Panther System

Aptima (Hologic) swabs were transported on ice and stored at 4°C for <30 days until testing by the Aptima Combo 2 for chlamydia and gonorrhea, by Aptima TV for trichomonas, and by Aptima MG for *Mycoplasma genitalium*.

#### Human Papilloma Virus Assay

The Digene HC2 vaginal brushes were transported on ice and stored at 4°C for <60 days until testing. The Roche Linear Array HPV Genotyping Test is a qualitative test that detects 37 human papillomavirus genotypes including 17 high risk types, 15 low-risk types, and 5 unknown-risk/probable-high-risk (pHR) types (Muñoz et al., 2003; de Villiers et al., 2004). The tests were performed according to the manufacturer's protocol (Woo et al., 2007), with broad-spectrum amplification and reverse line blot hybridization for genotype discrimination (Gravitt et al., 1998; Low et al., 2015).

#### CVS Characterization

Specimens were transported from the clinical location to the laboratory in a cooler on ice. Immediately upon return to the laboratory, the 50 mL conical tube containing the Softdisc was centrifuged at 1,000 RCF for 2 min to collect the CVS. The CVS was transferred to a 1.5 mL Eppendorf tube using a 50 μL Wiretrol (Drummond Scientific). The approximate sample volume was noted (average 0.25 ± 0.14 mL, range 0.1–0.5 mL). We were able to characterize 3 out of 4 Amsel's criteria for BV diagnosis, including: (i) pH >4.5, (ii) basic amine “fishy” odor upon mixing with 10% KOH (positive whiff test), and (iii) presence of clue cells in the wet mount. A sample had to meet all 3 criteria to be categorized as BV based on Amsel's criteria. CVS sample pH was measured using a Mettler Toledo EL20 pH meter with a micro-combination pH electrode MI-411 (Microelectrodes, Inc.). Slides were prepared for wet mount by rolling a swab coated in CVS on a standard microscope slide followed by the addition of 10 μL of normal saline and covered with a glass coverslip. The wet mount slide was observed for the presence of clue cells using differential interference contrast (DIC) microscopy. The whiff test was performed by dipping a cotton swab in the CVS, pipetting 100 μl of 10% KOH onto the swab, and using a gloved hand to waft air over to determine whether a fishy odor was produced. Another swab covered in CVS was rolled onto a second standard microscope slide and left to air dry for gram staining and Nugent scoring for diagnosis of BV (Nugent et al., 1991). For lactic acid measurements, ~10 μL of CVS was transferred to a preweighed Eppendorf tube to obtain the sample mass (6–20 mg). The CVS was diluted with 490 μL of normal saline and frozen at −20°C until performing the assay. The diluted samples were thawed and centrifuged at 1000 RCF for 5 min to pellet mucus solids. The supernatant was processed per manufacturer's instruction in a 96 well plate format using a D/L-lactic acid kit (R-Biopharm).

#### Multiple Particle Tracking to Assess HIV-1 Virion Mobility

Fluorescently labeled HIV-1 pseudoviruses were prepared as previously described (Nunn et al., 2015; Hoang et al., submitted). HIV virions (0.3 μL) were pipetted into 20 μL of undiluted CVS in a custom-made glass slide with a circular sample well, gently mixed, and immediately sealed with a glass coverslip. Twenty second videos of viral motion were recorded at room temperature using a Zeiss Axio Observer inverted epifluorescence microscope equipped with a 100x/1.46 NA oil-immersion objective and an EM-CCD camera (Evolve 512; Photometrics). The image resolution was 25 nm/pixel and sequential images were captured at a frame rate of 15 Hz. A minimum of 5 videos were collected per CVS sample. Virion trajectories were analyzed using automated MATLAB-based particle tracking software with a minimum of 16 frames (~1 s) of consecutive tracking as previously published (Suh et al., 2005; Lai et al., 2007; Schuster et al., 2015).

### 16S Sequencing and Analysis

#### DNA Extraction

BD Eswab fluid (150 μL) or 10 mg of CVS were resuspended in 180 μL of lysis buffer (1% Triton X-100, 20 mM Tris-HCl pH 8.0, 2 mM EDTA) with 20 mg/mL of lysozyme and incubated for 1 h at 37°C. Following lysozyme treatment, DNA was extracted using the DNeasy^®^ Blood and Tissue kit (QIAGEN^®^, Hilden, Germany), including the addition of 20 μL of proteinase K and incubation for 40 min at 56°C for pretreatment of gram-positive bacteria. Following DNA extraction, DNA concentrations were measured by NanoDrop.

#### DNA Amplification/Library Preparation

Library preparation was performed by the JHMI Deep Sequencing and Microarray Core at the Johns Hopkins Medical Institute according to the Illumina 16S metagenomic library preparation protocol (Illumina, San Diego, CA) with primers that amplified the V4 region of the 16S gene. Paired-end sequencing of the pooled library was performed with the MiSeq system (Illumina, San Diego, CA), generating 2 × 250 reads. Adaptor and barcode trimming was also performed by the JHMI Deep Sequencing and Microarray Core at the Johns Hopkins Medical Institute.

#### Sequence Processing

QIIME 1.9.1 was used to join paired reads, demultiplex samples, identify and filter out chimeras using USEARCH 6.1 (Edgar, 2010), perform open-reference OTU picking and make taxonomic assignments (Caporaso et al., 2010). Joining required a minimum of 15 base calls of overlap and <20% dissimilarity between matching sequences. Quality control during demultiplexing required 60% of base calls in a read to have a quality score above 17 with no more than 9 consecutive base calls below 17. The reference database used for OTU picking and taxonomic assignments is a custom reference database that combined an existing vaginal microbiome 16S database (Srinivasan et al., 2012) with the V4 regions of additional species represented in the VaHMP V1-V3 database (Fettweis et al., 2012).

#### Sequencing Data Analysis

Reads were rarefied in R (R 2017, R version 3.4.3) and k-means clustering was performed to assign samples to CSTs. To compare vaginal swab and CVS samples, Inverse Simpson Indices were calculated in R using the vegan (2.5–4) diversity command and a Bray-Curtis dissimilarity matrix, and NMDS analysis was performed using the vegan metaMDS command and a Bray-Curtis dissimilarity matrix. Stress of the NMDS analysis was 0.1725. PERMANOVA statistics were calculated using the vegan adonis command.

### Bacterial Culturing

*Lactobacillus crispatus* (BEI Resources strain EX533959VC06), *Lactobacillus jensenii* (ATCC strain 25258), *Lactobacillus iners* (BEI Resources strain UPII 60-B), *Lactobacillus gasseri* (ATCC strain 33323), and *Gardnerella vaginalis* (BEI Resources strain JCP7275) were all grown in either MRS (*L. crispatus, L. jensenii, L. gasseri*) or NYC III (*L. iners, G. vaginalis*) liquid broth. Bacteria grown in MRS were plated on MRS agar plates while bacteria grown in NYC III were plated on BBL™ Brucella Agar plates with 5% Sheep Blood with Hemin and Vitamin K1 (Becton Dickinson). Plates were incubated in anaerobic jars with GasPak™ EZ anaerobe container system (Becton Dickinson) at 37°C for 2–3 days before CFUs were counted (O'Hanlon et al., 2011).

### Quantitative PCR and Standard Curve Generation

#### Quantitative PCR

qPCR of vaginal samples and cultured bacteria was performed on Applied Biosystems, QuantStudio 3. Bacterial species-specific primers (Integrated DNA Technologies) for the 16S rRNA gene were used at a concentration of 100 nM (Supplementary Table 3) (Jespers et al., 2012). To standardize the qPCR, a fixed volume (2 μL) of DNA was used for cultured standards, CVS, and swab samples. The program started with an initial incubation at 95°C for 20 s, followed by 40 cycles with denaturing at 95°C for 1 s followed by anneal/extend at 60°C for 20 s. Data is shown for qPCR of CVS; the correlation coefficients for Ct values between CVS and swab was 0.97 for *L. crispatus*, 0.96 for *L. iners*, 0.96 for *L. jensenii*, 0.96 for *L. gasseri*, and 0.85 for *G. vaginalis*.

#### Species-Specific qPCR Standard Curve Generation

Bacteria were cultured overnight in liquid media (see above) then diluted 1:10 into fresh media and incubated for an additional 3–12 h to minimize the amount of dead bacteria. The subcultures were serially diluted 10-fold 5 times in triplicate. Serial dilutions were then plated as described above and used for DNA extraction. Following qPCR, the cycle thresholds (Ct) of the dilutions were plotted against the log of the CFU concentration for each species. Linear fitting provided an equation that was used to predict the CFU concentration in CVS based on the Ct for each species of bacteria.

#### Calculation of Lactobacilli Fraction

To calculate the fraction of a particular species of *Lactobacillus* using 16S sequencing data, the number of reads assigned to that species was divided by the total number of reads assigned to all *Lactobacilli*. To calculate the predicted fraction of a particular species of *Lactobacillus* using qPCR data, the predicted concentration (CFU/mL) for a particular species was divided by the total predicted CFU/mL for all *Lactobacilli* species.

### Statistical Analysis

PERMANOVA statistics were calculated on the Bray-Curtis dissimilarity command using the adonis command of the vegan package in R. Inverse Simpson indices were compared using the student's *t*-test. Correlation analysis and significance for qPCR standard curves and comparisons between specific *Lactobacillus* species representation from 16S rDNA sequencing and qPCR was performed in GraphPad Prism 8.1.0.

## Results

### Participant Demographics

Participant demographics are shown in Table 1. The median age of the participants was 26.5 yrs with a range of ages from 23 to 35 yrs. Two of the 20 (10%) of participants were Hispanic or Latino. The majority of participants were White (12/10, 60%) (Table 1).

### Notable Findings From Self-Reported Questionnaires

The participants answered questions about their sexual history, history of sexually transmitted infections, vaginal symptoms, and vaginal product use. A summary of key questionnaire data is provided in Table 1. Birth control usage was high, with participants reporting None (4/20, 20%), Condoms (2/20, 10%), Oral contraceptives (5/20, 25%), and copper or progestin intrauterine device (IUD) (9/20, 45%). Two participants (10%) reported BV in the past, one of which appeared to have active BV by Amsel's criteria (also noted below) and one who had the appearance of mixed bacteria on wet mount and gram staining. Prior human papilloma virus (HPV) infection was reported by one participant (5%). Prior yeast infection was reported by 12/20 participants (60%). One participant reported prior UTI. Prior chlamydia infection or treatment for potential exposure was reported by 4/20 participants (25%). Prior herpes infection was reported by 1 participant who was also on viral suppressive therapy (also noted below). Self-reporting of vaginal symptoms was rare and did not correlate well with other test results that were criteria for exclusion. For example, vaginal discharge (2/20, 10%) was reported by two participants with *Lactobacillus*-dominated microbiota, and not by the one participant that apparently had active BV. Vaginal product use was also rarely reported and did not correlate with other negative findings. Interestingly, the 1 participant that reported use of boric acid did not report prior incidence of BV, which is the situation where boric acid is most likely to be used. There was a wide range of reported total numbers of sexual partners (0–29). The number of new sexual partners during the past month was low (0–1), and the percentage of participants reporting that their current male partner was circumcised was high (15/20, 75%). All participants in the study reported no tobacco use and no prior pregnancies resulting in birth of a baby. Many of the participants also had some extent of medical records that were accessible to supplement the self-reported questionnaire answers. It was noted that one participant was on testosterone therapy, presumably as part of gender affirming therapy.

### Notable Findings From Testing

#### Blood Tests

As expected, a significant proportion (50%, 10/20) of the participants were CMV positive. Thus, while stool donors are typically not tested for CMV (Woodworth et al., 2017), we intend to ensure that CVS from CMV positive donors is used only in CMV positive recipients. Additionally, we found that 5/20 (25%) were positive for HSV-1 IgG, and two of these participants noted having oral cold sores in the past on follow-up. Out of an abundance of caution, we suggest that this be an exclusion criterion for potential VMT donors. Two participants out of 20 (10%) were HSV-2 IgG positive, where one was unaware of any past symptoms of infection and tested negative on the swab-based herpes NAT, and the other was on viral suppressive therapy. As expected, all participants (20/20, 100%) had rubella IgG and varicella zoster virus (VZV) IgG present due to past vaccination or exposure. Further, all participants tested were negative for rubella and VZV IgM, indicating there was no active infection. Likewise, 16/20 (80%) of participants were reactive for hepatitis A (HAV) IgG, but all participants tested were negative for HAV IgM. All participants (20/20) tested negative for current hepatitis B (HBV) and C (HCV) infections. One participant's bloodwork was indicative of microcytic anemia, which was consistent with a family history of thalassemia reported to the study team doctor on follow-up. While thassalemia does not have known impact on immunity or vaginal microbiota, for early investigations of VMT, a participant with thassalemia may be excluded under investigator discretion.

#### Swab Tests

We observed that there was not necessarily concordance between self-reporting of past yeast infection (12/20, 60%) and the presence of culturable yeast from a vaginal swab (6/20, 30%). Three of the participants that had culturable yeast did not report prior yeast infection, and 9 of the participants that reported past yeast infection did not have yeast reported after fungal culture. Regardless, all six participants with cultivable yeast (typically *Candida albicans*) did not have any symptoms of a yeast infection on follow-up. Bacteria species not considered to part of the normal urogenital flora were detected in 4/20 (20%) of participants. Two of these participants had culturable *Staphylococcus* bacteria but reported no symptoms of active infection on follow-up. One of the participants had light growth of numerous bacteria species including *Escherichia coli* and *Bacteroides* species, but reported that she dropped the swab on the floor when the study team doctor contacted for follow-up. One of the participants had moderate growth of *Corynebacterium* species on culture, which are typically innocuous. However, this participant was also the only participant (1/20, 5%) to test positive for *Mycoplasma genitalium*, and was instructed to see her physician on follow-up. All participants (20/20) tested negative for *Chlamydia trachomatis, Neisseria gonorrhoeae*, and *Trichomonas vaginalis*. One 1/20 participants (5%) tested negative for all strains of HPV, though another 6/20 (30%) had only “weak” or “very weak” positives that may warrant additional confirmatory testing. All participants (20/20, 100%) were negative for HPV16 and 18, the strains responsible for most HPV-related cancers. Given the intermittent nature of viral shedding, we suggest that vaginal swabs be collected with every donor CVS sample to test for HPV and herpes viruses.

#### Urine Tests

All participants tested negative for pregnancy, *Chlamydia trachomatis*, and *Neisseria gonorrhoeae*.

#### CVS

One participant did not report any history of BV, but the CVS sample characteristics were indicative of BV by fulfilling the 3 Amsel's criteria tested (positive whiff test, pH 4.62, visible clue cells/biofilm in wet mount). The presence of a polymicrobial bacteria community was confirmed by 16S rDNA sequencing (see below) and Nugent score (8–10). One participant had no notable exclusion criteria based on the questionnaire and screening tests, but the CVS itself appeared ovulatory (egg-white appearance, spinnbarkeit).

### Microbiota Communities Were Consistent Between Swabs and CVS

**Figure 1:**
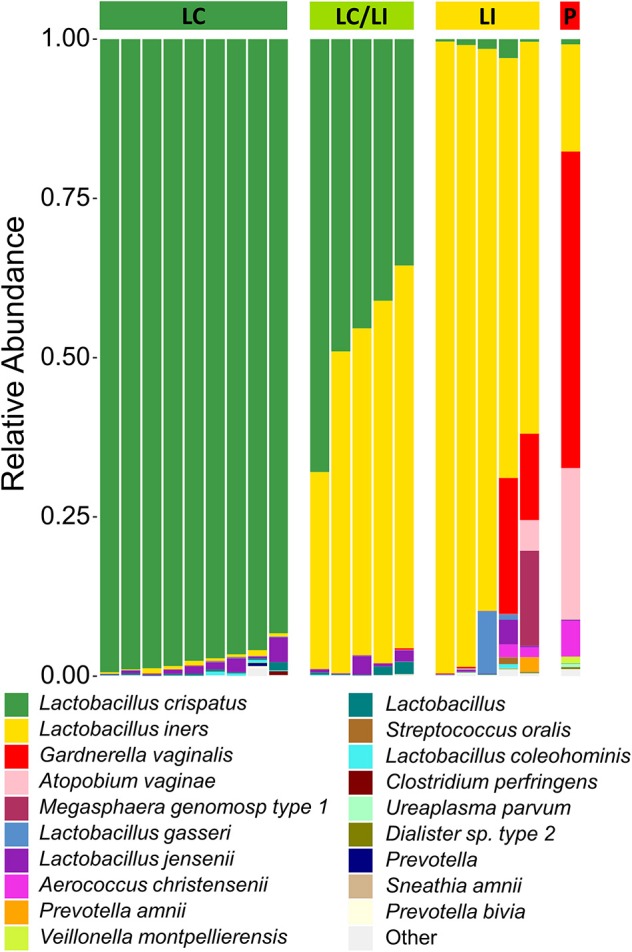
Stacked bar graph of vaginal bacteria phylotypes as determined by 16S rDNA sequencing of CVS. Samples are organized according to community state types (CSTs) as indicated by the colored bar on top of the graph. From left to right, green = *L. crispatus* (LC); yellow-green = *L. iners*/*L. crispatus* mix (LI/LC); yellow = *L. iners* (LI); red = polymicrobial (P). Each column represents an individual sample (*n* = 20 total). The height of each color indicates the relative abundance of a specific bacterial phylotype, as indicated in the legend. The top 19 abundant phylotypes are included, with all additional phylotypes summed and labeled as “Other”.

**Figure 2:**
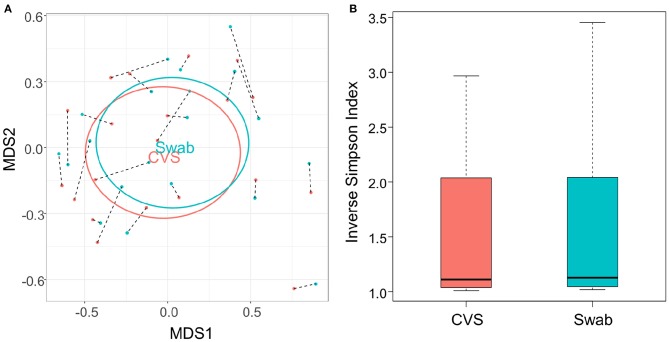
**(A)** NMDS plot of the Bray-Curtis dissimilarity matrix of CVS (pink) and swab (blue) samples. Black dashed lines connect each CVS sample with the swab obtained from the same donor. “CVS” and “Swab” are positioned on the centroids of the CVS and swab points, respectively, with standard deviations indicated by the ellipses (not significantly different, *p* = 0.986). **(B)** Boxplot of Inverse Simpson Indices calculated for CVS and swab samples (not significantly different, *p* = 0.7138).

There have been differing reports as to whether the sampling location and sampling method affects the composition of the vaginal microbiota (Kim et al., 2009; Virtanen et al., 2017). The Softdisc method described here collects material from the entirety of the cervicovaginal canal, which is then pooled together during centrifugation. Thus, we used 16S rDNA sequencing to characterize the vaginal microbiota communities for paired CVS and vaginal swab samples to determine whether the results would be similar. As shown in Figure 1, we found that 19/20 (95%) of the participants had CVS dominated by *Lactobacillus* spp., which was consistent with testing negative for BV based on the three Amsel's criteria characterized. CVS samples clustered into four distinct community state types (CSTs), three of which were predominated by *Lactobacillus spp*. (*L. crispatus, L. iners*, or a *L. crispatus*/*L. iners* mix). When comparing sequencing of DNA extracted from CVS samples and vaginal swabs, paired CVS samples and vaginal swabs were not discernible from each other using NMDS analysis (Figure 2A) and applying PERMANOVA did not reveal a significant difference between the distribution of the CVS samples or vaginal swabs (*p* = 0.986). The paired samples from each participant clustered into concordant CSTs and did not show a significant difference in diversity, as measured by the inverse Simpson Index (*p* = 0.7138) (Figure 2B).

### Standardized qPCR Correlates With the Relative Abundance of Specific Bacterial Species

**Figure 3:**
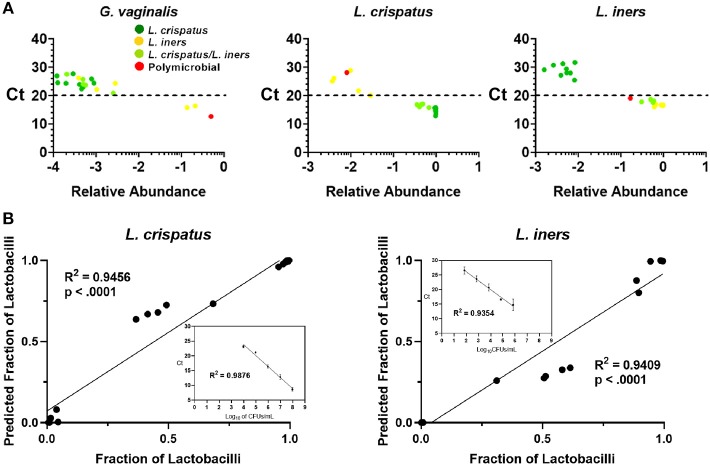
**(A)** Individual CVS samples according to their relative species abundance, as obtained from 16S rDNA sequencing, and their Ct determined by qPCR using species specific primers for *G. vaginalis, L. crispatus*, and *L. iners*. Individual data points are color coded for each group based on sequencing. Dashed lines indicate Ct = 20, our suggested threshold. **(B)** Individual CVS samples according to the predicted fraction of *L. crispatus* or *L. iners* relative to the combined lactobacilli concentrations determined by qPCR compared to that obtained from the 16S rDNA sequencing. Insets show the standard curve used to estimate the concentration of the indicated species and calculate the predicted fraction by qPCR.

Because 16S rDNA gene sequencing is a high-throughput approach, samples are typically batched together in large numbers. For the purpose of screening CVS samples to rapidly determine individual donor and sample eligibility, we sought to determine whether qPCR could be used to provide rapid compositional information that was reflective of sequencing. Using species-specific primers for *L. crispatus, L. jensenii, L. iners, L. gasseri*, and *G. vaginalis*, we performed 5 qPCR reactions on DNA extracted from each CVS sample. By comparing the qPCR results with the relative abundance obtained from the 16S sequencing, we determined that in the case of *G. vaginalis*, a Ct cutoff of 20 readily distinguished three samples that had >10% relative abundance (red bars in the last three columns in Figure 1) from samples with low relative abundance of *G. vaginalis* (Figure 3A). Furthermore, Ct <20 reliably predicted dominance by Lactobacillus species (Figure 3A, Supplementary Figure 1A), though not necessarily the relative abundance of each species in mixed samples. Therefore, to allow rapid predictive CST classification before 16S rDNA sequencing, we generated qPCR standard curves from laboratory strains of *L. crispatus, L. jensenii, L. iners*, and *L. gasseri* to relate Ct values with the concentration of colony forming units (CFU) per volume across four logs in concentration in serial dilutions. Strong linearity was observed in all four species over four logs in bacteria concentration (insets in Figure 3B, Supplementary Figure 1B). The comparison of the predicted fractional representation based on qPCR and the fractional representation based on the 16S rDNA sequencing is shown for the dominant species (*L. crispatus, L. iners*) in Figure 3B and for the minor species (*L. jensenii, L. gasseri*) in Supplementary Figure 1B. In all four cases, we observed strong correlations between the predicted relative abundance based on qPCR and the relative abundance based on 16S rDNA sequencing.

### CVS Physicochemical Properties Correlate With the Presence of Lactobacillus

**Figure 4:**
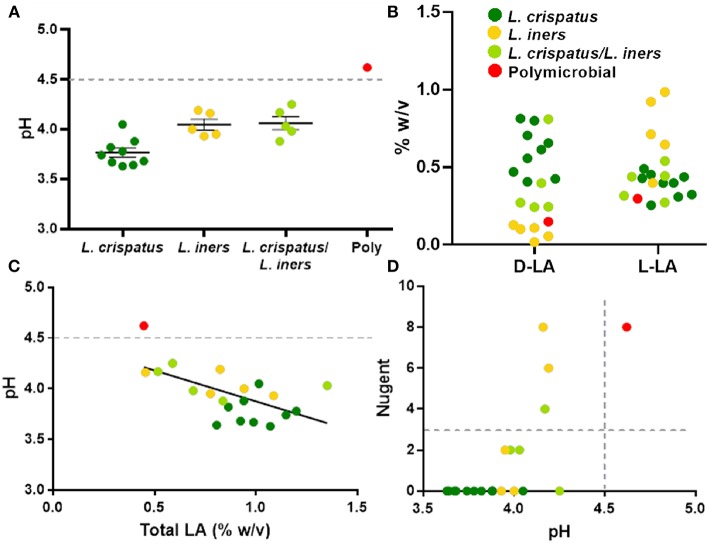
**(A)** Individual CVS sample pH grouped based on 16S rDNA sequencing. The group mean ± SEM is shown. The gray dotted line corresponds to pH 4.5, which is the clinical cutoff for BV according to Amsel's criteria. **(B)** Concentrations of D- and L-isomers of lactic acid (LA) in CVS. Individual data points are color coded for each group based on sequencing. **(C)** CVS pH as a function of total (D + L) LA content. The gray dotted line corresponds to pH 4.5, which is the clinical cutoff for BV according to Amsel's criteria. Individual data points are color coded for each group based on sequencing. Linear regression line shown, *r*^2^ = 0.37, *p* = 0.004. **(D)** Nugent score as a function of pH. Individual data points are color coded for each group based on sequencing. The gray dotted lines correspond to the threshold for Nugent score considered negative for BV (≤3) and the clinical cutoff for BV according to Amsel's criteria (pH 4.5).

The protective role of the lactic acid and low pH of the vagina appears to be important for maintaining vaginal health (Olmsted et al., 2005; Aldunate et al., 2013; Hearps et al., 2017; Tachedjian et al., 2017), suggesting that the physicochemical characteristics of the CVS may be more important than the particular species present. As shown in Figure 4A, dominance by *L. crispatus* is generally associated with lower pH. In comparison, samples dominated by *L. iners* or a mixture of *L. crispatus* and *L. iners* had a slightly higher average pH, though still below the clinical cutoff for BV (>4.5, gray dotted line). Indeed, only the one sample that was polymicrobial had a pH above 4.5 (Figure 4A). We also found the relative concentrations of the D- and L- isomers of lactic acid (D-LA, L-LA) were reflective of the bacteria present in the CVS sample. Namely, there were relatively higher levels of D-LA compared to L-LA in CVS samples dominated by *L. crispatus*, where the opposite was true in samples dominated by *L. iners* (Figure 4B). For the samples that were a mixture of *L. iners* and *L. crispatus*, the concentrations for D-LA and L-LA were more similar, with the exception of one sample that had higher D-LA concentrations (Figure 4B), which may reflect the metabolic contributions of both species. The total concentration of lactic acid (D + L) generally increased with decreasing pH, meaning that *L. crispatus*-dominated samples typically had higher total LA concentrations, though there also were *L. iners*-dominated samples and *L. crispatus*/*L. iners* mix samples within the cluster (Figure 4C). We further found that the ability of the CVS to adhesively trap fluorescently labeled HIV-1 virions correlated with the *Lactobacillus* spp. present. *L. crispatus*-dominated CVS consistently trapped virions (Supplementary Figure 2A), supporting the mucosal barrier function to pathogens. However, virion mobility was more variable in *L. iners* and *L. iners*/*L. crispatus* mix CVS samples, where some samples trapped virions and other samples allowed for permissive diffusion of virions (Supplementary Figure 2A). Generally, virion mobility increased with increasing CVS pH, with the transition being relatively sharp around pH 4.2 (Supplementary Figure 2B). Thus, as pH is relatively easy to measure in real time and is an indicator of dominance by *Lactobacillus* spp. and the amount of lactic acid produced, we suggest a pH cutoff of ≤4.2 for use in VMT. Similarly, we found that a cutoff pH of ≤4.2 also correlated well with scoring negative for BV by Nugent (≤3) (Figure 4D). A few samples that were *Lactobacillus*-dominated by sequencing and pH <4.5 had high Nugent scores in the intermediate (4–6) or BV (7–8) range. Two of these samples were dominated by *L. iners* but had significant amounts of *Gardnerella vaginalis* (Nugent scores 6 and 8). Based on these data, we suggest that Nugent score ≤2 is sufficient to identify *Lactobacillus*-dominated samples with high lactic acid content and low pH suitable for VMT.

### Potential Eligibility Findings

The full lists of clinical and laboratory tests performed can be found in Supplementary Tables 1, 2, respectively. FDA testing requirements indicate that a potential donor of leukocyte-rich cells or tissues that is not sexually intimate with the recipient must be tested for HIV (types 1 and 2), HBV, HCV, *Treponema pallidum*; HTLV (types 1 and 2), CMV, *Chlamydia trachomatis*, and *Neisseria gonorrhea*. We propose that for VMT, a potential donor candidate should also be tested for HAV, HSV-1/2, VZV, EBV, rubella virus, *Toxoplasma gondii*, HPV, *Trichomonas vaginalis*, and *Mycoplasma genitalium*, cultivable yeast/fungi, and cultivable bacteria. The test list employed here included redundant serological testing and vaginal swab-based testing of herpes viruses (HSV-1/2, VZV). All active infections should be excluded. Evidence of past exposure to CMV, VZV, and rubella (including vaccination) should be matched between donors and recipients. We suggest that the evidence of past exposure to HSV-1 or HSV-2 should be an exclusion criterion. The presence of cultivable yeast in the absence of symptoms of an active yeast infection and/or evidence of yeast growth in the wet mount preparation need not be an exclusion criterion unless the participant indicates having had a history of yeast infections (>1 in the past). We suggest similar guidelines that only UTI occurring on more than 1 occasion be grounds for exclusion. The HCT/P guidelines stipulate chlamydia infection is only an exclusion if it has occurred in the prior 12 months, though we suggest that infections occurring >12 months ago should be considered along with other history and could potentially be grounds for exclusion at investigator discretion upon review. The presence of cultivable bacteria that are not considered “normal” urogenital flora (terminology used by the pathology laboratory) would result in exclusion. We suggest that self-reported product use need not be an exclusion criteria alone, but that donor participants should be instructed to avoid insertion of any vaginal products (including tampons) during the entire CVS collection period. Our results suggest that there is not a clear rationale for setting a maximum limit for number of sexual partners for inclusion as a CVS donor. However, we suggest that donor participants should be required to avoid participating in vaginal, rectal, or oral intercourse, as well as use of sex toys, digital penetration, etc. during the entire CVS collection period. As the effects of exogenous hormones on the vaginal microbiota are not well-studied, we determined that this would be an exclusion criteria for participating as a VMT donor. Further, while the procedures for participation as a donor do not likely pose any risk to pregnant women, we propose that the potential impacts of hormones and other factors on the vaginal microbiome during pregnancy be grounds for exclusion. Ovulatory CVS or CVS collected at the time of menses would not be ideal for transplant due to low bacteria density and elevated pH, but would not exclude a donor from providing additional CVS samples in the future.

For the purposes of screening a potential donor's vaginal microbiota composition, our results suggest that qPCR can be used to perform rapid characterization of the relative abundances of the four common *Lactobacillus* species that is predictive of the relative abundance by 16S rDNA sequencing. We prefer the use of CVS isolated with the menstrual cup collection approach to a vaginal swab for this purpose, because with isolated CVS, sample mass can be standardized. In contrast, the amount of CVS collected with a vaginal swab is not known or easily standardized, which would affect the threshold Ct value between samples. Of course, use of this qPCR approach would require individual labs to generate standard curves using reference bacteria and standardization using individual instrumentation and reagents. Using our qPCR instrument and a standard mass of 10 mg CVS, we found that Ct < 20 (>10% relative abundance by 16S sequencing) for *G. vaginalis* should be an exclusion criterion. Similarly, dominance by *Lactobacillus* species was predicted by Ct < 20. Together with Nugent scoring, pH measurement, and lactic acid measurements, dominance by *Lactobacillus* species can be quickly confirmed in individual CVS samples. Further, all samples should still be characterized by confirmatory 16S rDNA sequencing, which is standard in the field. Also, we would suggest that a standard plating method and counting of CFUs should be used to determine the overall *Lactobacillus* “dose.” While the HIV mobility assay is informative and correlative with other CVS physicochemical properties, the equipment and materials required are specialized and need not be considered a standard screening approach.

Based on the test results and exclusion criteria discussed above, we found that 7/20 (35%) of our participants may be eligible for CVS donation as part of a future VMT study. However, we anticipate that the actual success rate for participation as a CVS donor in a clinical trial will be much lower than 35% for several reasons. This small pilot study used an abbreviated screening questionnaire, as described in the Materials and Methods. The participants were selected from a pool of participants from the study team's prior clinical studies, increasing the likelihood that they would have *Lactobacillus*-dominated vaginal microbiota and fulfill the donor criteria. Of note, the majority of the participants were White or Asian, which is consistent with the observation that White and Asian women in the U.S. are less likely to have BV (Allsworth and Peipert, 2007; Fettweis et al., 2014; Peebles et al., 2019). However, efforts should be made to recruit a racially/ethnically diverse donor pool, as the potential impact of race/ethnicity on the success of VMT is unknown. Additionally, the more stringent cut-off values suggested based on the participants in this study (Nugent ≤ 2, pH ≤ 4.2) may need to be adjusted for a larger and more racially/ethically and/or geographically diverse donor pool. Further, the “universal” donor approach would typically consist of an initial full blood, swab, and urine screening like that described here, followed by a 30–60 day period of sequential CVS sample collections (excluding menses and ovulation if not using hormonal contraceptives). Out of an abundance of caution, we propose that donors be willing to abstain from vaginal intercourse and vaginal product use during the full duration of CVS collection, which was not an eligibility criterion for this small pilot study. Although not performed here, we also suggest that every donor CVS sample be tested for the presence of semen with a test like the ABAcard P30 test for the forensic identification of semen. We further propose that due to the intermittent nature of viral shedding (Wald et al., 1995; Phipps et al., 2011; Gravitt and Winer, 2017), vaginal swabs be collected to test for herpes viruses and HPV at the time of every CVS sample. After collection of the last CVS sample, the donor should go through the full blood, swab, and urine testing procedure again after a quarantine period of ~30 days. It is likely that this series of additional testing will result in additional screening failures.

## Discussion

Although the first modern literature report of fecal microbiota transplant (FMT) was documented in 1958 (Eiseman et al., 1958), it was not until 2013 that the first randomized clinical trial for treating recurrent *C. difficile* infection with FMT was reported (van Nood et al., 2013). Although the study did not involve blinding, it was considered to unequivocally represent the potential of FMT as a safe and effective treatment (Sampath et al., 2013). The prior year, the FMT process was greatly streamlined by demonstrating that equivalent efficacy could be achieved with (i) stool from standard volunteer donors rather than donors identified by the patient, and (ii) stool frozen prior to use (Hamilton et al., 2012). These findings paved the way for various stool banks around the country that screen potential donors and collect, process, and freeze stool for distribution to clinical providers. OpenBiome, a non-profit that reportedly shipped over 43,000 treatments since starting its service in 2013, was an early innovator in developing thorough screening and quarantining procedures for frozen, ready-to-use stool preparations to increase patient access and safety^1^. The development of the FMT field is an obvious source of inspiration for initiating study of other forms of microbiota transplantation, such as VMT. Thus, our goal here was to conceptualize a universal VMT donor screening process that is uniquely suited to what we understand about vaginal microbiota and sexually transmitted infections. For example, unique considerations for VMT donor participants include the number of sexual partners, practice of certain sexual behaviors and frequency, and use of vaginal products. Further, any history of sexually transmitted infections should be grounds for exclusion. The stringency of the universal donor screening process inherently limits the availability of eligible donor participants, which is why OpenBiome reports only 3% of their participants screened are deemed eligible for stool donation (Dubois et al., 2015)^1^. It is possible that if VMT were to be successful, a similar model for donor screening with CVS banking and distribution could be implemented. Further, the development of a rationally designed biotherapeutic product using clinical outcome data from VMT studies would be a logical next step, both from a safety perspective as well as the much larger potential patient population. The trajectory of the FMT field is similarly informative in this regard, and will aid in accelerating the development of other microbiota-based therapeutic strategies.

Much of what we understand about the dynamics of vaginal microbiota was learned in studies that employed vaginal swabs, self-collected or physician collected, as the means for isolating bacteria (Aagaard et al., 2012; Gajer et al., 2012; DiGiulio et al., 2015). Other studies have reported the physicochemical properties of undiluted CVS or cervicovaginal lavage fluids, which gives us added indirect measures of bacteria composition and some aspects of metabolic function (Lai et al., 2009a,b, 2010; O'Hanlon et al., 2011; Chappell et al., 2014; Nunn et al., 2015). However, we and others have begun characterizing both the CVS and the composition of the vaginal microbiota to begin to correlate community structure and function (Nunn et al., 2015; Hoang et al., submitted). Here, we further demonstrate that the community structure is strikingly similar whether using a vaginal swab or an aliquot of the CVS as the biological matrix for DNA extraction, which supports the use of vaginal swabs as a reliable and valid representation of the native mucosal environment. Similar to what was described for *Lactobacillus* bacteria in culture, we found that the relative concentrations of D- and L-lactic acid were reflective of the *Lactobacillus* spp. present in the CVS (Witkin et al., 2013). We also found that *L. crispatus*-dominated CVS tended to have higher total lactic acid content, lower pH, and more effective immobilization of fluorescently-labeled HIV virions, which is consistent with prior observations made by our group and others (Nunn et al., 2015; O'Hanlon et al., 2019). Our data shown here also suggested that using a CVS pH cut-off of ≤4.2 and a Nugent score cut-off of ≤2 would be most suitable for VMT, which is slightly more stringent than the criteria defined for categorizing samples as negative for BV (Amsel et al., 1983; Nugent et al., 1991).

The procedures and data shown herein are not intended to be wholly inclusive of every consideration for screening potential donors for VMT. Further, to operate under an investigational new drug application (IND), there are additional considerations for Chemistry, Manufacturing, and Controls (CMC). In the case of stool transplants, which were known to be efficacious for treating recurrent *C. difficile* prior to regulation by the FDA, we have yet to identify relevant measures of potency. In contrast to a small molecule drug or biologic, we do not know the key “active” component in stool, and thus cannot quantify the concentration or activity of that component. Further, the clinical success of using processed stool with 10% glycerol added prior to freezing (a standard approach for freezing bacteria stocks) was reported without characterization of the stool before and after freezing (Hamilton et al., 2012; Satokari et al., 2015). Many studies since have looked at the effect of processing and storage on the stability of particular bacteria or the overall bacteria community (Costello et al., 2015; Fouhy et al., 2015), though there is still not a general consensus on what is important for a successful clinical outcome. In the case of VMT, donor CVS is largely considered a monoculture of one *Lactobacillus* species, and thus, lends itself to performing characterization of the colony forming units (CFU) per unit volume before and after freezing. We can also determine the effect of cryoprotectants and other media on the *Lactobacillus* viability, which is an area of active study in our group. Of note, however, although we can directly characterize the “potency” (concentration in CFU/mg) of the *Lactobacillus*, it is also possible that the lactic acid, mucins, or other components of the host environment could play a role in the potential success of the transplantation process. While vaginal microbiota can be considered relatively monomicrobial, there is also evidence here and described by others that more than one species of *Lactobacillus*, such as *L. crispatus* and *L. iners*, can cohabitate (Ravel et al., 2011; Gajer et al., 2012). Further, although other species may be in the minority, it has yet to be determined whether the overall community structure with major and minor players is important. We anticipate that the trajectory of VMT will likely follow that of FMT, wherein there are many parallel efforts in academia and industry to determine what minimum cultivable components can be manufactured to produce uniform, standardized products with similar therapeutic efficacy as stool (Kelly et al., 2015; Hoffmann et al., 2017; Ott et al., 2017).

In addition to intensive and thorough screening of potential donor participants for VMT, it is also important to consider testing of potential recipient participants. An inherent risk of the VMT procedure is transmission of a sexually transmitted pathogen. For various reasons associated with participant safety and for determining the overall safety of the VMT procedure, the baseline infection status of the potential recipient should be known. Thus, potential recipients should be screened similarly to potential donor participants, but the inclusion and exclusion criteria would differ. In addition to the obvious consideration that BV would be an exclusion for donors but not for recipients, it is also feasible that a controlled herpes infection or HPV would not necessarily be an exclusion criterion for potential recipient participants. Because of the transient and intermittent nature of viral shedding, potential recipient participants would ideally be screened at multiple time points prior to undergoing the VMT procedure to maximize detection of asymptomatic viral shedding of HSV-1, HSV-2, and HPV. Further, samples for testing should be taken immediately prior to the VMT procedure, because the results will help interpretation of any notable findings that may occur during recipient follow-up, including whether they may be attributable to the donor CVS. As the field of VMT is in its infancy, ensuring safety and tolerability of the procedure is of paramount importance in the first clinical studies. Various standardized adverse event grading systems are available, such as the *Division of AIDS (DAIDS) Female Genital Grading Table for Use in Microbicide Studies* for local vaginal/reproductive tract adverse events. Further, follow-up testing of recipient participants can also determine whether a potential shift in vaginal microbiota composition impacts future risk of acquiring sexually transmitted infections. It is also conceivable that as VMT may be explored for other indications, additional follow-up considerations may be timing of childbirth and birth outcomes, or recurrence of conditions such as urinary tract infection or yeast infection.

Another important aspect to consider is the VMT procedure itself. As we describe, the donor CVS was collected using a non-absorbent polymer-based cup, allowing for collection of undiluted material by centrifugation. However, similar to FMT, dilution with fluid such as sterile saline would ease loading into a device for application and administration to the recipient. Thus, we anticipate that similar to FMT, dosing would be determined by the ratio of sample mass to diluent fluid volume. This is in contrast to the dosing approach for probiotic products, where the concentration of CFU/dose can be standardized during the manufacturing procedure—with CVS donation, the mucus itself may be as important as the bacterial colony count. However, in the case of CVS, we observe a wide range of CFU/mg between participants, which is also reflective of the functional differences of the dominant bacteria. As such, when working with CVS from individual donors, it is not straightforward to standardize the dose based on the concentration of CFU. This is perhaps particularly true for *Lactobacillus* species found in CVS, because when they are the predominant species, they are present in lower numbers of CFU than the total number of CFU when the community is polymicrobial (Hill, 1993). Indeed, the ability of lactobacilli to colonize and compete out other bacterial species in CVS does not appear to be directly dependent on their concentration.

In the discussion of how microbiota transplantation must be regulated differently than standard drugs and biologics, it is worth noting that VMT and vaginal microbiota transfer from mother to babies born by Cesarean section, or “vaginal seeding,” uses the same “drug.” However, the nature of the screening procedures and the handling of the CVS are quite different, and thus, VMT has many more parallels to FMT than vaginal seeding. For example, the notion of universal donors cannot apply to vaginal seeding, as the donor must be the mother. Thus, screening failure rules out the possibility of conducting the procedure at all. Further, because of the relevant time constraints, the vaginal seeding procedure also rules out the possibility of performing thorough testing of the CVS at the time of collection (1 h before Cesarean section) and quarantining in the frozen state until test results are known. The CVS collection procedure for vaginal seeding is also quite different and requires no subsequent processing; absorbent gauze or swabs are used to “soak up” the CVS for direct transfer to the baby's skin after birth. Thus, the definition of the “dose” is also different, in that a single dose would just be the amount of CVS that is collected in the gauze while it is left in the mother's vagina. Finally, the use of the term “transfer” rather than “transplant” reflects the fact that, by definition, transplanted material is placed in the same area of the body from which it was obtained. In the case of vaginal seeding, the CVS is swabbed onto the baby's mouth, nose, and skin in order to mimic the process of passing through the vaginal canal during birth. In contrast, VMT is a true transplant of CVS from the vagina of the donor to the vagina of the recipient.

## Conclusion

This project represents a multidisciplinary effort to establish a protocol for comprehensive screening and characterization of donor CVS for the purpose of VMT, including a parallel rigorous screening process for potential recipients of VMT. The limitations of this pilot study include the small sample size and selection of participants from past study participant pools known to the study team to have optimal microbiota, which would not be generalizable to larger populations. It is possible that the requirement for sexual abstinence throughout the CVS sample collection period, or the requirement for providing multiple sequential self-collected swabs and CVS samples intensively over a month-long period, may constrain enrollment in future studies. The stringency of our exclusion criteria for donors, motivated by the ethical and clinical imperative to avoid potential transmission of pathogens, is both necessary and challenging from the perspective of recruitment. However, once a donor has been identified, using this protocol, as a safe donor, she could ideally donate CVS on multiple appropriately screened occasions; the idea of a “super-donor” with no identified past or current infections and with favorable *Lactobacillus*-dominated microbiota is one that should be explored and is of potential high impact to the project and the field. It is certain that as the field of microbiota transplant expands, unique regulatory questions and dynamic scientific, ethical, and clinical considerations will continue to arise (Hoffmann et al., 2017). These should be met with equally dynamic innovative approaches that incorporate optimized human safety considerations while recognizing the potential value of microbiota transplantation in addressing BV and its varied adverse impacts on human health.

## Data Availability

The raw data supporting the conclusions of this manuscript will be made available by the authors, without undue reservation, to any qualified researcher. As of submission, the raw sequencing data is not publicly available, but public links or the data itself will be supplied on request.

## Author Contributions

LE, EW, CG, PG, EF, CH, RC, and JC contributed conception and design of the study. LE wrote the first draft of the manuscript and KD, SB, and EW wrote sections of the manuscript. KD, SB, FZ, HZ, TH, LE, CG, and PG conducted experiments, oversaw staff testing of clinical specimens, and/or collected clinical specimens. EW, EF, and CH oversaw the clinical study and coordinated clinical activities. KD, SB, FZ, HZ, TH, PG, EW, AA, and LE performed data analysis and/or data interpretation. All authors contributed to the manuscript revision, read, and approved the submitted version.

### Conflict of Interest Statement

The authors declare that the research was conducted in the absence of any commercial or financial relationships that could be construed as a potential conflict of interest.

## Footnotes

^1^OpenBiome (2019). Available online at: https://www.openbiome.org/impact (accessed March 09, 2019).
