# Supplementary material for: Conceptual Design of a Universal Donor Screening Approach for Vaginal Microbiota Transplant
Source: Front Cell Infect Microbiol. 2019 Aug 28;9:306. doi: 10.3389/fcimb.2019.00306 (PMC6722226; doi:10.3389/fcimb.2019.00306)
Supplement: Supplementary file 1 [file Data_Sheet_1.docx]

Supplementary Material

**Supplementary Table 1.** Clinical tests

| **JHMI MEDICAL LABORATORIES** | | | |
| --- | --- | --- | --- |
| **Soft code** | **Test** | **Submeasures** | **Readout** |
| **Blood** | | | |
| EBVM | EBV IgM VCA | N/A | present or not present |
| TOXOG | Toxoplasma gondii IgG | N/A | present or not present |
| RUBIG | Rubella IgG | N/A | present or not present |
| HSVG | HSV-1 and -2 IgG | HSV-1 IgG | present or not present |
|  |  | HSV-2 IgG | present or not present |
| VZVIG | VZV IgG | N/A | present or not present |
| SERIG | Quantitative immunoglobulins | Serum IgG | 751 - 1560 mg/dL |
|  |  | Serum IgA | 82 - 453 mg/dL |
|  |  | Serum IgM | 46 - 304 mg/dL |
| CBCDF | CBC with differential | White blood cell count | 4.5 - 11.0 K/cc mm |
|  |  | Red blood cell count | 4.00 - 5.20 M/cu mm |
|  |  | Hemoglobin | 12.0 - 15.0 g/dL |
|  |  | Hematocrit | 36.0 - 46.0% |
|  |  | Mean corpuscular volume | 80.0 - 100.0 fL |
|  |  | Mean corpuscular Hgb | 26.0 - 34.0 pg |
|  |  | Mean Corpus HgB conc | 31.0 - 37.0 g/dL |
|  |  | RBC distribution width | 11.5 - 14.5% |
|  |  | Platelet count | 150 - 350 K/cc mm |
|  |  | Mean platelet volume | 9.2 - 12.7 fL |
|  |  | NRBC number | 0.00 - 0.01 K/cu mm |
|  |  | Lymphocytes % | 24.0 - 44.0% |
|  |  | Monocyte % | 2.0 - 11.0% |
|  |  | Neutrophil % | 40.0 - 70.0% |
|  |  | Immature gran % | 0.0 - 1.0% |
|  |  | Eosinophil % | 1.0 - 4.0% |
|  |  | Basophil % | 0.0 - 2.0% |
|  |  | Neutrophil number | 1.50 - 7.80 K/cu mm |
|  |  | Lymph number | 1.10 - 7.80 K/cu mm |
|  |  | Monocyte number | 0.10 - 1.20 K/cu mm |
|  |  | Eosinophil number | 0.12 - 0.30 K/cu mm |
|  |  | Immature gran number | 0.00 - 0.05 K/cu mm |
| C4C3L | CD4 absolute | CD3+ lymphocytes | 51 - 91% |
|  |  | CD4+ lymphocytes | 32 - 68% |
|  |  | Absolute CD4+ lymphocytes | 458 - 1344 cells/cu mm |
| MEBVQ | EBV PCR on plasma | EBV Viral Load, plasma | <50 copies/mL |
|  |  | EBV Log Value, plasma | <1.70 copies/mL |
| HIVSR | HIV-1/2 Ag/Ab w/Reflex | HIV-1/2 Ag/Ab summary | reactive/non-reactive |
|  |  | HIV-1/2 Ag/Ab screen | reactive/non-reactive |
| Q0876 | Rubella IgM | N/A | Reference range <20.00 |
| Q0562 | VZV IgM | N/A | Reference range <= 0.90 |
| HAV | Hepatitis A IgM | N/A | reactive/non-reactive |
| HAABT | Hepatitis A antibody, total | N/A | reactive/non-reactive |
| **Urine** | | | |
| UHCG | Pregnancy (urine HCG) | N/A | Negative/indeterminate/positive |
| **Vaginal Swabs** | | | |
| MH12V | HSV PCR | HSV-1 NAT | detected/not detected |
|  |  | HSV-2 NAT | detected/not detected |
|  |  | VZV NAT | detected/not detected |
| MYCUL | Fungal culture | N/A | Fungal species recovered |
|  | Aerobic/anaerobic bacterial culture | Bacterial Gram stain | Presence of leukocytes, gram staining |
| CXMNG |  |  |  |
|  |  | Bacteria aerobic/anaerobic culture | Bacterial species recovered, antibiotic susceptibility |
| **MEMORIAL BLOOD CENTERS** | | | |
| **BLOOD** | **Test** | **Submeasures** | **Readout** |
|  | Hepatitis B | HBsAg (no reflex) | positive/negative |
|  |  | GS-HBsAg EIA | reactive/non-reactive |
|  |  | Anti-HBc Total series | positive/negative |
|  |  | Anti-HBc Total (IgG + IgM) | reactive/non-reactive |
|  | MPX 2.0 series | NAT HBV (Hep B) | reactive/non-reactive |
|  |  | NAT HCV (Hep C) | reactive/non-reactive |
|  |  | NAT HIV | reactive/non-reactive |
|  | Hepatitis C | Anti-HCV no reflex series | positive/negative |
|  |  | Anti-HCV EIA | reactive/non-reactive |
|  | HIV | Anti-HIV-1,2 plus O (no reflex) | positive/negative |
|  |  | Anti-HIV-1,2 plus O EIA | reactive/non-reactive |
|  | HTLV | Avioq HTLV-1/II EIA series | positive/negative |
|  |  | Avioq HTLV-1/II EIA | reactive/non-reactive |
|  | Syphilis | Syphilis MHA-TP (no reflex) | positive/negative |
|  |  | Syphilis MHA-TP | reactive/non-reactive |
|  | CMV | CMV total (no reflex) series | positive/negative |
|  |  | CMV total (IgG + IgM) | positive/negative |
|  | West Nile Virus | WNV PCR series | positive/negative |
|  |  | WNV PCR | reactive/non-reactive |
| **URINE** | Chlamydia | GP Chlamydia series | positive/negative |
|  |  | GP Chlamydia trachomatis RNA | positive/negative |
|  | Gonorrhea | GP Gonorrhoeae Series | positive/negative |
|  |  | GP GN Gonorrhoeae RNA | positive/negative |

**Supplementary Table 2.** Laboratory tests

| **LABORATORY TESTS** | |
| --- | --- |
| **Test/Measurement** | **Readout** |
| **Aptima vaginal swab** | |
| Chlamydia trachomatis | Positive/negative |
| Neisseria gonorrhoeae | Positive/negative |
| Trichomonas vaginalis | Positive/negative |
| Mycoplasma genitalium | Positive/negative |
| **Digene HC2 vaginal brush** | |
| Roche Linear Array | Positive/weak positive/very weak positive/negative |
| **CVS sample** | |
| Estimated volume | 0.1-1 mL |
| Consistency | Watery, stretchy, normal |
| Color | White, yellow, brown |
| Nugent | 0-10 |
| pH | 0-14 |
| whiff test | Positive/negative |
| Wet mount | Clue cells, neutrophils, yeast, sperm cells, bacteria (rods, spherical) etc. |
| qRT-PCR/16S rRNA sequencing | Relative abundance of bacteria species, clustering based on dominant *Lactobacillus* or polymicrobial |

**Supplementary Table 3**. Table of forward and reverse primer sequences.

| Bacteria | Forward sequence 5’-3’ | Reverse sequence 5’-3’ |
| --- | --- | --- |
| *L. crispatus* | AGCGAGCGGAACTAACAGATTTAC | AGCTGATCATGCGATCTGCTT |
| *L. iners* | GTCTGCCTTGAAGATCGG | ACAGTTGATAGGCATCATC |
| *L. jensenii* | AAGTCGAGCGAGCTTGCCTATAGA | CTTCTTTCATGCGAAAGTAGC |
| *L. gasseri* | AGCGAGCTTGCCTAGATGAATTTG | TCTTTTAAACTCTAGACATGCGTC |
| *G. vaginalis* | TTACTGGTGTATCACTGTAAGG | CCGTCACAGGCTGAACAGT |


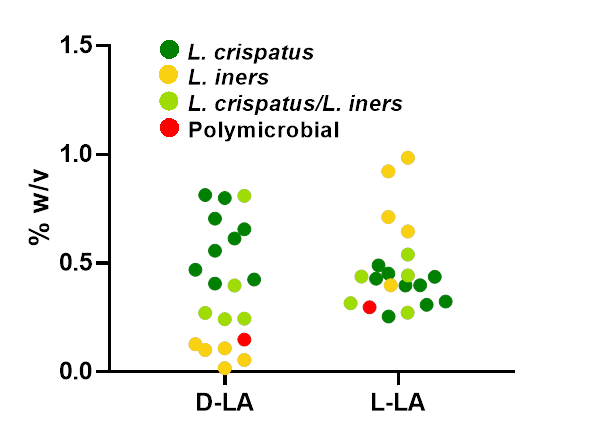

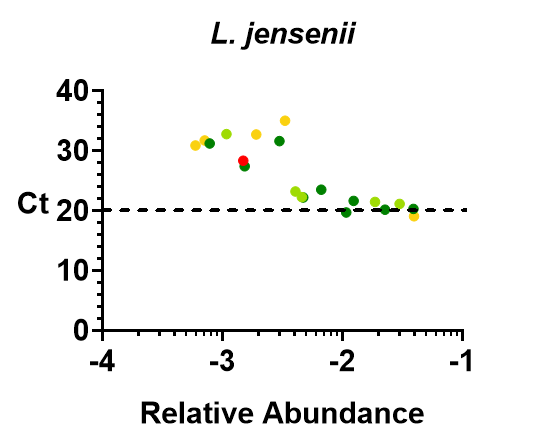

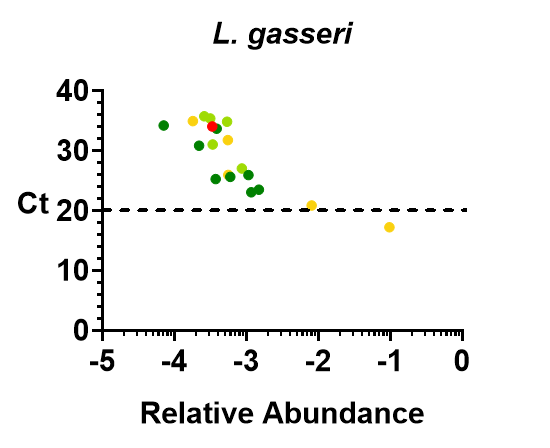


**A**


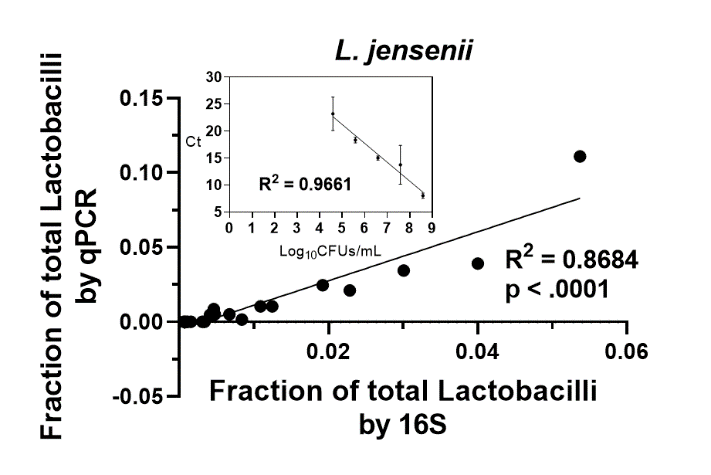

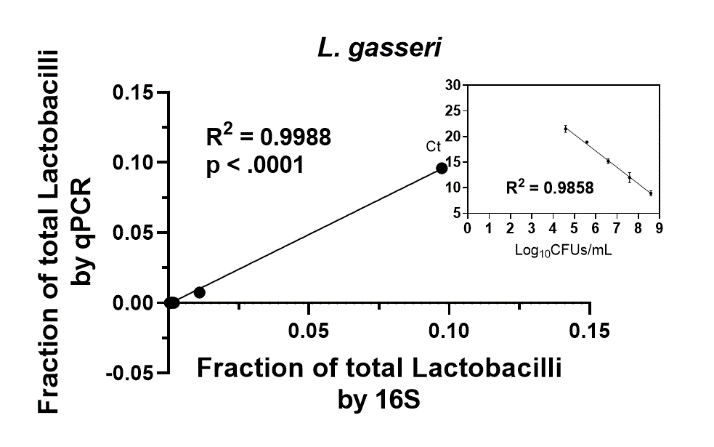


**B**

Supplementary Figure 1. (A) Individual CVS samples according to their relative species abundance, as obtained from 16S rDNA sequencing, and their Ct determined by qPCR using species specific primers for *L. jensenii*, and *L. gasseri*. Individual data points are color coded for each group based on sequencing. Dashed lines indicate Ct = 20, our suggested threshold. (B) Individual CVS samples according to the fraction of *L. jensenii* or *L. gasseri* of the combined Lactobacilli abundance as obtained from the 16S rDNA sequencing and the predicted fraction as determined from qPCR. Insets show the standard curve used to estimate the concentration of the indicated species and calculate the predicted fraction.


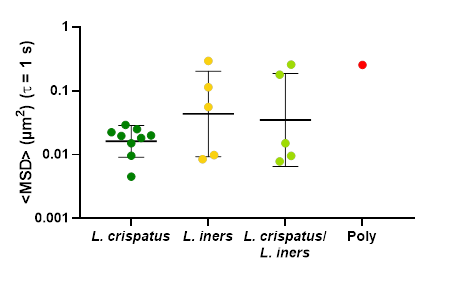

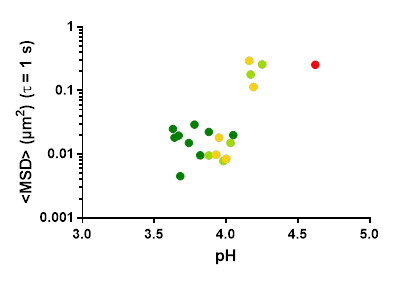


Supplementary Figure 2. (A) HIV virion ensemble-averaged mean square displacement (<MSD>) at a time scale ($\tau$) of 1 s, where each data marker represents an individual CVS sample (n = 20). Samples are organized by group assignments based on 16S rDNA sequencing. Data represented as geometric mean and geometric mean standard deviation. (B) HIV virion <MSD> plotted as a function of CVS pH.

**A**

**B**
